# Seroprevalence of Avian Influenza A(H5N6) Virus Infection, Guangdong Province, China, 2022

**DOI:** 10.3201/eid3004.231226

**Published:** 2024-04

**Authors:** Yang Wang, Chunguang Yang, Yong Liu, Jiawei Zhang, Wei Qu, Jingyi Liang, Chuanmeizi Tu, Qianyi Mai, Kailin Mai, Pei Feng, Wenjing Huang, Zhengshi Lin, Chitin Hon, Zifeng Yang, Weiqi Pan

**Affiliations:** The First Affiliated Hospital of Guangzhou Medical University, Guangzhou, China (Y. Wang, C. Yang, J. Zhang, W. Qu, J. Liang, C. Tu, Q. Mai, K. Mai, W. Huang, Z. Lin, Z. Yang, W. Pan);; Guangzhou National Laboratory, Guangzhou (Y. Wang, C. Yang, C. Hon, Z. Yang);; Guangzhou Kingmed Center for Clinical Laboratory Co., Ltd., Guangzhou (Y. Liu);; Macau University of Science and Technology, Macau, China (P. Feng, C. Hon, Z. Yang, W. Pan)

**Keywords:** Avian influenza, A(H5N6), China, seroprevalence, influenza, respiratory infections, viruses, zoonoses

## Abstract

In 2022, we assessed avian influenza A virus subtype H5N6 seroprevalence among the general population in Guangdong Province, China, amid rising numbers of human infections. Among the tested samples, we found 1 to be seropositive, suggesting that the virus poses a low but present risk to the general population.

The highly pathogenic avian influenza A virus subtype H5, identified in Guangdong Province, China, in 1996, has evolved into multiple distinct phylogenetic clades and undergone reassortment events (*1*). In 2014, a new clade (2.3.4.4) that included influenza A(H5N6) virus emerged in Asia and has caused both epizootic and zoonotic cases worldwide (*2*). As of August 1, 2023, a total of 86 human cases of H5N6 infection have been reported globally; 40 (46.5%) have resulted in death (*3*). Most cases were reported in China, and 1 case was reported in Laos (*3*). An increase in the number of H5N6 human infections during 2021 and 2022 has been observed, reaching a total of 55 cases, exceeding the cumulative total number of the reported H5N6 human infections in the preceding years (Figure, panel A). This sudden upsurge has consequently raised concerns over a higher risk for H5N6 transmission.

**Figure:**
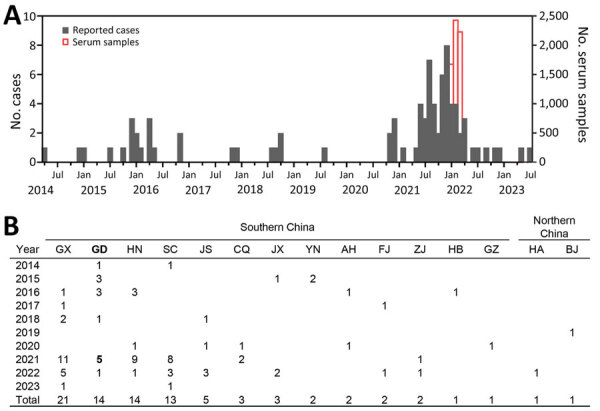
Collection timepoints and locations of 6,363 persons from whom residual serum samples were collected in Huizhou and Dongguan cities, Guangdong Province, China, plotted against the temporal and spatial distribution of human infection with influenza A virus subtype H5N6 in China as a whole. A) Temporal distribution of 85 human infections with H5N6 in China during 2014–2023 and collection timepoints of 6,363 residual serum samples. Scales for the y-axes differ substantially to underscore patterns but do not permit direct comparisons. B) Geographic distribution of 85 human H5N6 infections in China by province, municipality, or autonomous region, as of August 1, 2023. The numbers represent the confirmed cases of infection in each area. Guangdong Province (boldface), the site of the seroprevalence study, reported all 5 local H5N6 cases in 2021 within Dongguan (n = 2) and Huizhou (n = 3) cities, where the residual serum samples were collected. AH, Anhui; BJ, Beijing; CQ, Chongqing; FJ, Fujian; GD, Guangdong; GX, Guangxi; GZ, Guizhou; HA, Henan; HB, Hubei; HN, Hunan; JS, Jiangsu; JX, Jiangxi; SC, Sichuan; YN, Yunnan; ZJ, Zhejiang.

Previous studies have indicated a higher prevalence of human infections with H5 viruses, according to serologic evidence, compared with the number of World Health Organization–confirmed cases (*4*). A shortage of serologic surveillance studies focusing on human H5N6 infections in the general population exists (*5*,*6*). To better assess the risk for H5N6 infections during the 2021–22 wave, we conducted a cross-sectional serologic study during January–March 2022 (Figure, panel A) in Dongguan and Huizhou cities in Guangdong Province. The cities were the epicenters of H5N6 human infections in 2021 (Figure, panel B). Given the unclear seroprevalence of H5N6 virus in the general population, we used an estimated H5N1 seropositivity rate of 1.2% (*4*) for our sample size calculation, targeting a 95% CI and a precision of 0.006. Assuming a dropout rate of 15%, we calculated that a sample size of 6,012 in the general population would be required. This study was approved by the ethics committee of the First Affiliated Hospital of Guangzhou Medical University (ethics approval no. 2016-78).

We excluded poultry workers and patients with oncologic diseases, hematologic malignancies, or immunocompromising conditions from our study. The patients who reported respiratory symptoms or diseases were not excluded and represented a small fraction of the sample pool (46 [0.72%]). We collected serum samples from 6,363 participants at 72 local hospitals and physical examination centers across Dongguan and Huizhou cities, ensuring a broad regional representation. Of the participants, most were outpatients (4,284 [67.33%]); the remaining participants were hospitalized patients (699 [10.99%]) or persons undergoing routine physical examinations (1,380 [21.69]). The median age of participants was 41 years (25th–75th percentile 29–55 years). Of the 6,363 samples, 42.2% (2,685) were from men and 57.8% (3,678) from women; 53.4% (3,401) of samples were from Huizhou (Table). We screened the residual serum samples by using a hemagglutination inhibition (HI) assay against a recombinant H5N6 virus derived from A/Huizhou/1/2021(H5N6) (2.3.4.4b subclade), according to previously published studies (*7*). We confirmed 15 serum samples with an HI titer >10 by microneutralization assay, as described previously (*7*). We defined a seropositive result for H5N6 as having both HI and microneutralization titers >20 (*6*). Among those samples, we identified 1 confirmed seropositive specimen, collected on March 15, 2022, from a 22-year-old woman with an HI titer of 1:20 and an microneutralization titer of 1:80. We also identified 1 suspicious specimen, collected on March 1, 2022, from a 3-year-old boy with an HI titer of an 1:10 and an microneutralization titer of 1:40 (Appendix Table). Neither participant had a reported history of influenza-like illness before serum collection. All other samples were seronegative.

**Table:** Demographic characteristics of 6,363 persons from whom serum samples were collected for influenza A virus subtype H5N6 titer testing, Huizhou and Dongguan, Guangdong Province, China, January–March 2022*

Demographic characteristic	Value
Age group, y	
0–14	566 (8.9)
15–24	531 (8.3)
25–54	3633 (57.1)
55–64	768 (12.1)
>65	865 (13.6)
Median age, y (25th–75th percentile)	41 (29–55)
Sex	
M	2,685 (42.2)
F	3,678 (57.8)
Location	
Huizhou	3,401 (53.4)
Dongguan	2,962 (46.6)

*Values are no. (%) except as indicated.

The seroprevalence of H5N6 infection in humans varies across different regions and time (*5*,*6*). In this cross-sectional study, we identified 1 seropositive serum sample among 6,363 residual serum samples collected from the general population in Huizhou and Dongguan, China, during January–March 2022. The seroprevalence in the general population was lower than that among poultry workers, which can reach up to 2.0% (*6*). This difference in seroprevalence suggests a lower risk factor for H5N6 infection in the general population compared with poultry workers, consistent with the poultry-to-human transmission route of H5N6 virus (*8*). Of note, the participant with a seropositivity against H5N6 virus did not report a history of influenza-like illness, indicating that the virus can also cause mild or asymptomatic infections. Our findings underscore the necessity of enhancing surveillance for H5N6 virus, especially in poultry workers or persons with poultry contact history, because of their higher risk for exposure.

One limitation of our study is that the exclusion of poultry workers and the lack of poultry contact history information limited our capacity to assess risk for these specific groups. In addition, our focus on Guangdong Province may not reflect other regions’ epidemiologic profiles. We used cross-sectional residual serum samples instead of paired serum samples collected before and after the 2021–22 A(H5N6) wave. The paired serum sample approach, although more challenging, could better identify recent infections by tracking antibody titer changes.

Appendix Additional information about seroprevalence of avian influenza A(H5N6) virus infection, Guangdong Province, China, 2022.

## References

[1] Smith GJ, Donis RO; World Health Organization/World Organisation for Animal Health/Food and Agriculture Organization (WHO/OIE/FAO) H5 Evolution Working Group. Nomenclature updates resulting from the evolution of avian influenza A(H5) virus clades 2.1.3.2a, 2.2.1, and 2.3.4 during 2013-2014. Influenza Other Respir Viruses. 2015;9:271–6. 10.1111/irv.12324 25966311 PMC4548997

[2] Jeong S, Otgontogtokh N, Lee DH, Davganyam B, Lee SH, Cho AY, et al. Highly pathogenic avian influenza clade 2.3.4.4 subtype H5N6 viruses isolated from wild whooper swans, Mongolia, 2020. Emerg Infect Dis. 2021;27:1181–3. 10.3201/eid2704.203859 33754986 PMC8007304

[3] Center for Health Protection. Avian influenza report, volume 19, number 30. 2023 Aug 1 [cited 2023 Aug 1]. https://www.chp.gov.hk/files/pdf/2023_avian_influenza_report_vol19_wk30.pdf

[4] Wang TT, Parides MK, Palese P. Seroevidence for H5N1 influenza infections in humans: meta-analysis. Science. 2012;335:1463. 10.1126/science.1218888 22362880 PMC4160829

[5] Ma MJ, Zhao T, Chen SH, Xia X, Yang XX, Wang GL, et al. Avian influenza A virus infection among workers at live poultry markets, China, 2013–2016. Emerg Infect Dis. 2018;24:1246–56. 10.3201/eid2407.172059 29912708 PMC6038753

[6] Quan C, Wang Q, Zhang J, Zhao M, Dai Q, Huang T, et al. Avian influenza A viruses among occupationally exposed populations, China, 2014–2016. Emerg Infect Dis. 2019;25:2215–25. 10.3201/eid2512.190261 31742536 PMC6874249

[7] Guan W, Qu R, Shen L, Mai K, Pan W, Lin Z, et al. Baloxavir marboxil use for critical human infection of avian influenza A H5N6 virus. Med. 2024;5:32–41.e5. 10.1016/j.medj.2023.11.001 38070511

[8] Zhu W, Li X, Dong J, Bo H, Liu J, Yang J, et al. Epidemiologic, clinical, and genetic characteristics of human infections with influenza A(H5N6) viruses, China. Emerg Infect Dis. 2022;28:1332–44. 10.3201/eid2807.212482 35476714 PMC9239879

