# Supplementary material for: Seroprevalence of Avian Influenza A(H5N6) Virus Infection, Guangdong Province, China, 2022
Source: Emerg Infect Dis. 2024 Apr;30(4):826–8. doi: 10.3201/eid3004.231226 (PMC10977835; doi:10.3201/eid3004.231226)
Supplement: Appendix — Additional information about seroprevalence of avian influenza A(H5N6) virus infection, Guangdong Province, China, 2022. [file 23-1226-Techapp-s1.pdf]

Article DOI: https://doi.org/10.3201/eid3004.231226

EID cannot ensure accessibility for supplementary materials supplied by authors. Readers who have difficulty accessing supplementary content should contact the authors for assistance.

Seroprevalence of Avian Influenza A(H5N6) Virus Infection, Guangdong Province, China, 2022

Appendix

Appendix Table. Hemagglutination inhibition and microneutralization titers against A(H5N6) virus from serum samples collected between January and March 2022, in Guangdong Province, China, with a hemagglutination inhibition titer ≥10. Case #9 resulted in suspicion for positivity of a confirmed A(H5N6) case but was not definitive. Case #13 was confirmed positive for A(H5N6) infection.

		Hemagglutination Inhibition titers		Microneutralization titers	
No.	Sample ID	Test 1	Test 2	Test 1	Test 2
#1	22A45	10	10	<10	<10
#2	22E199	20	20	<10	<10
#3	22F174	40	20	<10	<10
#4	22H73	20	10	<10	<10
#5	22H65	10	10	<10	<10
#6	22H61	10	20	<10	<10
#7	22H62	10	10	<10	<10
#8	22H63	10	20	<10	<10
**#9**	**22J778 (the suspicious case)**	**10**	**10**	**40**	**40**
#10	22J930	10	20	<10	<10
#11	22K31	20	10	<10	<10
#12	22K400	40	20	<10	<10
**#13**	**22K1660 (the confirmed case)**	**20**	**20**	**80**	**80**
#14	22L521	10	10	<10	<10
#15	22M488	20	10	<10	<10
